# Effect of Nano-Modified Recycled Wood Fibers on the Micro/Macro Properties of Rapid-Hardening Sulfoaluminate Cement-Based Composites

**DOI:** 10.3390/nano15130993

**Published:** 2025-06-26

**Authors:** Chunyu Ma, Liang Wang, Yujiao Li, Qiuyi Li, Gongbing Yue, Yuanxin Guo, Meinan Wang, Xiaolong Zhou

**Affiliations:** College of Civil Engineering & Architecture, Qingdao Agricultural University, Qingdao 266109, China; 20242113002@stu.qau.edu.cn (C.M.);

**Keywords:** recycled wood fiber, rapid-hardening sulfoaluminate cement, nano-silica modification, silane coupling agent, hydration properties

## Abstract

Recycled wood fiber (RWF) obtained through the multi-stage processing of waste wood serves as an eco-friendly green construction material, exhibiting lightweight, porous, and high toughness characteristics that demonstrate significant potential as a cementitious reinforcement, offering strategic advantages for environmental protection and resource recycling. In this study, high-performance sulfoaluminate cement (SAC)-RWF composites prepared by modifying RWFs with nano-silica (NS) and a silane coupling agent (KH560) were developed and their effects on mechanical properties, shrinkage behavior, hydration characteristics, and microstructure of SAC-RWF composites were systematically investigated. Optimal performance was achieved at water–cement ratio of 0.5 with 20% RWF content, where the KH560-modified samples showed superior improvement, with 8.5% and 14.3% increases in 28 d flexural and compressive strength, respectively, compared to the control groups, outperforming the NS-modified samples (3.6% and 8.6% enhancements). Both modifiers improved durability, reducing water absorption by 6.72% (NS) and 7.1% (KH560) while decreasing drying shrinkage by 4.3% and 27.2%, respectively. The modified SAC composites maintained favorable thermal properties, with NS reducing thermal conductivity by 6.8% through density optimization, whereas the KH560-treated specimens retained low conductivity despite slight density increases. Micro-structural tests revealed accelerated hydration without new hydration product formation, with both modifiers enhancing cementitious matrix hydration product generation by distinct mechanisms—with NS acting through physical pore-filling, while KH560 established Si-O-C chemical bonds at paste interfaces. Although both modifications improved mechanical properties and durability, the KH560-modified SAC composite group demonstrated superior overall performance than the NS-modified group, providing a technical pathway for developing sustainable, high-performance recycled wood fiber cement-based materials with balanced functional properties for low-carbon construction applications.

## 1. Introduction

With the acceleration of urbanization, a large amount of construction solid waste generated after the demolition of old houses is in urgent need of recycling [1]. The resource utilization of construction solid waste is conducive to the green and low-carbon transformation of the construction industry and can reduce negative impacts on the environment [1]. The preparation of concrete, mortar or other building materials after the pretreatment, strengthening, and screening of construction solid waste [2,3,4,5] has become a new trend in the green and low-carbon development of the construction industry. A certain amount of discarded wood exists in the demolition solid waste of wooden buildings, brick and wooden buildings [6], and waste furniture. According to the statistics, waste wood accounts for 7.5% and 11% of demolition waste and construction waste, respectively. If not properly treated, it occupies substantial land resources and may release unpleasant odors and organic compounds during decomposition, leading to environmental pollution. The rational utilization and repurposing of waste wood have become a crucial approach to alleviating the supply–demand imbalance of timber resources and achieving sustainable wood resource management. After the crushing and screening of waste wood, different particle size grades of regenerated wood fiber are obtained. Recycled wood fibers have the characteristics of light mass and high toughness [7,8] and also are more beneficial to the environment than steel fibers [9,10], basalt fibers [11,12,13], glass fibers [14,15], and carbon fibers [16,17], which are incorporated into cementitious materials for subsequent degradation. Incorporation of treated waste wood fibers into cementitious materials can bring new performance advantages to cementitious materials [18,19] and also alleviate the pressure of waste wood recycling [20,21,22].

In recent years, many researchers at home and abroad have carried out research work related to the incorporation of wood fibers into cementitious materials. Zhu et al. [23] found that when the wood fiber content was increased to 3.0%, the flow of mortar was significantly reduced. The 28 d compressive strength of mortar increased significantly when a 1.0% volume fraction of wood fiber was added, and the 72 d self-shrinkage of mortar decreased significantly. Zhou et al. [24] investigated the thermal and mechanical properties of lightweight thermal insulation mortar prepared from waste recycled wood fibers. The results showed that, in terms of thermal insulation performance, a reduction in the cement–wood ratio would enhance the thermal insulation performance of the mortar. When the cement–wood ratio is 3.0, its 28 d compressive and flexural strength increased compared to the control group, which indicates that the addition of wood fiber can improve the mechanical properties and increase the toughness and crack resistance of mortar. Liu et al. [25] investigated the mechanical and shrinkage properties of alkali-excited mortar mixed with recycled wood powder and indicated that when the wood powder yield reached 6%, alkali-excited mortar had a higher compressive strength and fracture strength than the control group. The compressive strength of the recycled mortar decreased, but it was still higher than the compressive strength of the control mortar. The incorporation of wood fiber also significantly reduced the shrinkage of the mortar. Kittipong et al. [26] studied and compared the microstructure of waste wood fiber mortar under different curing conditions; their microanalysis showed that autoclave-curing resulted in hydration products in a crystalline form, which were formed during the curing process. Tong et al. [27] investigated lightweight bamboo fiber-reinforced thermal insulation mortar’s mechanical properties and thermal insulation properties; the results of bamboo fiber incorporation and wood fiber incorporation results were similar, both reduced the density and mechanical strength of the mortar, and to a certain extent increased the thermal insulation properties of the mortar. The above findings indicate that waste wood fiber-reinforced cementitious materials achieve simultaneous density reduction and flexural strength improvement while addressing premium timber shortages, demonstrating high feasibility and practical application potential. However, many studies have conducted life cycle assessments on biological fibers [28,29,30], indicating that although biological fibers are mainly derived from natural raw materials, the performance improvement of pure regenerated biological fibers relative to natural fibers is not significant due to the lack of obvious optimization of fiber pretreatment on a laboratory scale. Further environmental advantages come from the enhanced performance ensured by biological fiber modification, which can significantly save on materials and reduce costs compared to using pure biological fibers, with the same performance.

Although the incorporation of recycled wood fibers can improve several properties of mortar, due to defects, such as high water absorption, poor interfacial bonding, and the many microcracks of the recycled wood fibers themselves [31,32,33,34], it is necessary to further modify the recycled wood fibers to improve the performance of cementitious materials. Lin et al. [35] carried out a study on the physical properties of modified waste wood fiber-enhanced mortar, showing that the alkali solution treatment and silica fume significantly improved the tensile strength and crack resistance of the waste wood fiber mortar. Chen et al. [36] studied the performance of silane-modified wood fiber-filled EPDM bio-composites, showing that the tensile strength and modulus of EPDM biocomposites treated with the modification increased. Bastidas et al. [37] investigated the influence of alkaline NaOH solution on the mechanical properties of wood fiber-reinforced mortar; compared with the control group, the mechanical properties of the alkali-treated wood fiber mortar increased by an average of 25% and showed a better interfacial bonding structure between the cement matrix and the treated wood fibers. However, traditional alkali solution modification has defects, such as reduced fiber strength, fragile interface bonding, and easy formation of hydrophilic pores [38,39], while silica fume modification is prone to aggregation, hinders hydration reactions, and has uncontrollable shrinkage performance, all of which seriously limit its promotion and application. Ghamarpoor et al. [40] found that rubber nanocomposites modified by nano grafting have excellent mechanical properties and water separation performance, which can improve the crosslinking density with polymers and enhance the separation efficiency between organic matter and water molecules. This has positive implications for reducing the absorption of hydration water by wood fibers. Huang et al. [41] indicated that the “pozzolanic reaction” and “nucleation effect” of grafted nano silica accelerate the hydration of cement paste, producing more hydrated calcium silicate (C-S-H), exhibiting excellent heat resistance and structural stability under high-temperature conditions. Xiang et al. [42] prepared cationic polymer-grafted nano silica using the atom transfer radical polymerization (ATRP) reaction. Due to the synergistic effect of cationic polymer and nano silica, the crosslinking density between silica particles and hydration products increased; the cement paste exhibited high chloride ion penetration impedance and enhanced compressive strength. Zhang et al. [43] The grafting of SiO_2_ layer can promote the production of additional hydration products in cement paste, resulting in tighter adhesion and stronger interfacial bonding between the grafted polymer and the cement matrix. Radhakrishnan et al. [44] analyzed the microstructure and life cycle of the new engineered cement-based composite materials. Results from the tensile tests revealed that using natural wood fibers in an engineered cementitious composites mix aided in achieving significant tensile strength and failure strain levels.

Therefore, the novelty of this study lies in the modification of waste-derived recycled wood fiber (RWF) using NS solution and KH560 [45]. By the interface Si-O-C chemical bonding of KH560 and the filling of nanoscale pores in NS, revealing their distinct action mechanisms, this study successfully incorporated a high content of RWF into sulphoaluminate cement (SAC) composites, collectively enhancing the composite’s mechanical properties, durability, and thermal performance. This approach led to the development of a novel green building material that combines environmental friendliness with excellent comprehensive performance, providing a technical pathway for low-carbon building applications. Notably, KH560 modification demonstrated stronger advantages in interfacial chemical bonding and shrinkage suppression.

## 2. Experimental Procedure

### 2.1. Raw Materials

#### 2.1.1. Cementitious Material

In this study, rapid-hardening sulfoaluminate cement (SAC) is used as the cementitious material, with a strength grade of 42.5, provided by Tangshan Polar Bear Building Materials Co., Ltd., Tangshan, China. The density of the SAC is 2950 kg/m^3^, the initial setting time is 27 min, the final setting time is 57 min, and the specific surface area is 380 m^2^/kg. The detailed XRF chemical composition of SAC is shown in Table 1.

#### 2.1.2. Fine Aggregate

**Figure 1:**
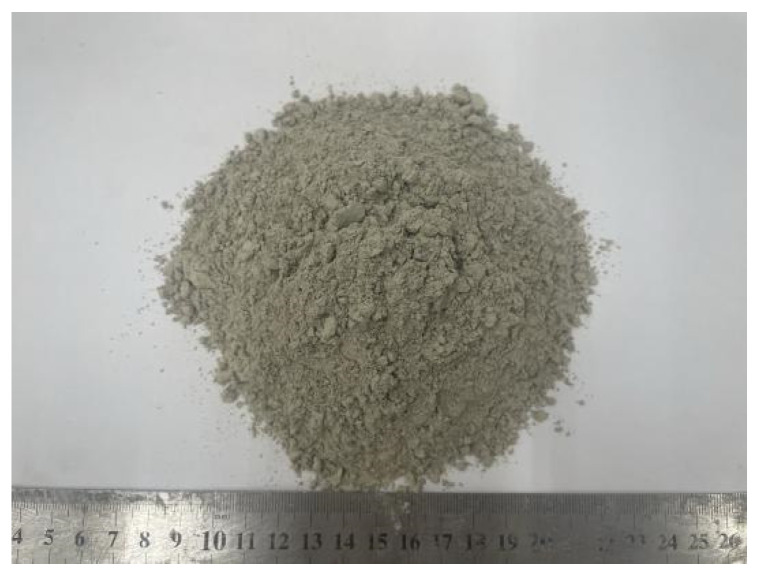
Rapid-hardening sulphoaluminate cement.

**Figure 2:**
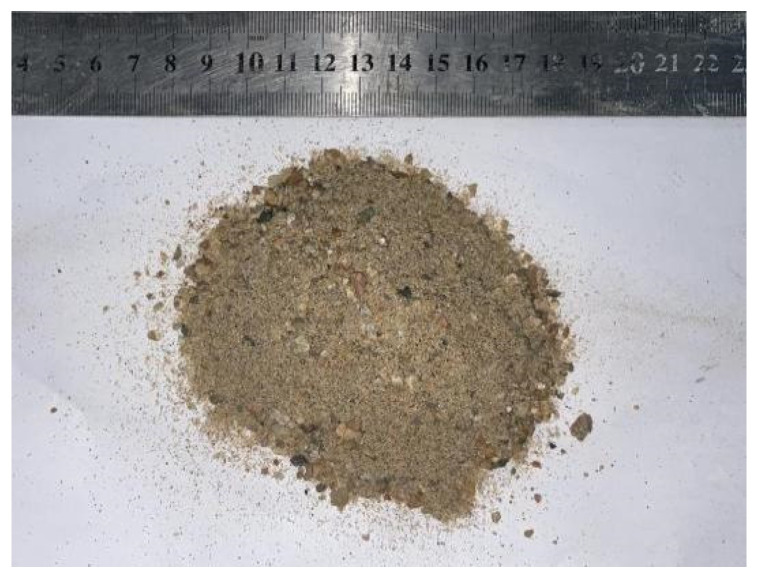
Natural river sand.

River sand with a fineness modulus of 2.7 is selected as a fine aggregate (provided by Qingdao Lvfan Recycled Building Materials Co., Ltd., Qingdao, China). The physical indexes of the fine aggregate are shown in Table 2, its bulk density is 1465 kg/m^3^, apparent density is 2600 kg/m^3^, crushing index is 11.2% and water absorption rate is 1.5%. The appearances of SAC and river sand are shown in Figure 1 and Figure 2, respectively.

#### 2.1.3. Recycled Wood Fibers

**Figure 3:**
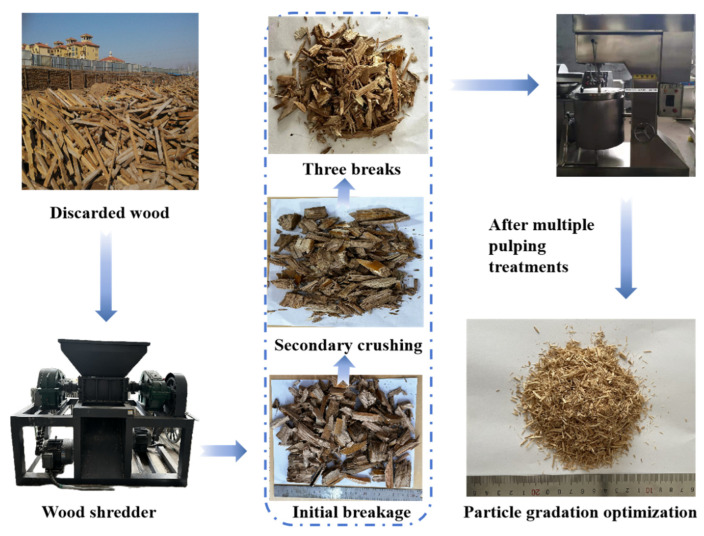
Flowchart for the preparation of recycled wood fibers (RWFs).

The waste wood used in this study was recycled from the demolition of wooden buildings, brick and wood buildings, and furniture. The recycled wood was mechanically crushed and pulped by a wood crusher several times; the waste wood was processed into 20–50 mm wood chips and pieces to improve the subsequent processing efficiency. Wood fibers are then separated through friction in a refiner, followed by vibration screening to remove oversized fibers through a 4.75 mm standard square sieve before undergoing drying treatment to obtain the recycled wood fiber (RWF) used in the test. The specific flowchart of the preparation of the RWF is shown in Figure 3. The particle size distribution of the RWF is mainly between 0.6 and 2.36 mm, the length of the RWF is between 1–3 mm, the density is 964.8 kg/m^3^, the water absorption rate is 99.8%, and the water content is 4.2%.

#### 2.1.4. Modification of RWFs

**Figure 4:**
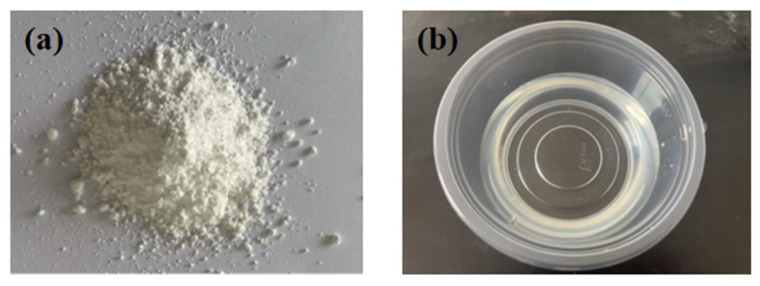
Modifier for recycled wood fiber: (**a**) nano-silica (NS); and (**b**) silane coupling agent KH560.

Recycled wood fibers may exhibit issues such as hydrophilicity, high water absorption, and poor compatibility with cementitious matrix materials. Therefore, in this study, 1 wt% nano-silica (NS) and 1 wt% silane coupling agent (KH560) are employed to modify the RWF and further investigate the effect of modified RWFs on the performance development of rapid-hardening sulphoaluminate cement-based materials. The incorporation of NS can promote cement hydration, while NS-modified RWFs can improve the surface roughness of fibers and enhance the interfacial adhesion between RWFs and cementitious materials. Accordingly, this research utilizes nano-silica (NS) with 99.5% purity and a particle size of 50 ± 5 nm, provided by Shandong Keyuan Biochemical Co., Ltd., Jinan, China for RWF modification. The KH560 silane coupling agent, serving as a surface modifier, was supplied by Nanjing Shuguang Chemical Group Co., Ltd., Nanjing, China. This KH560 modifier presents as a colorless transparent liquid with a boiling point of 290 °C, a refractive index of 1.426–1.428nD25, and a specific gravity of 1.065–1.072dD25. Silane coupling agent modification can improve the flexural strength and interlaminar shear strength of RWF materials, thereby influencing the macroscopic properties of cement-based composites. The modifier for the RWF is shown in Figure 4a,b.

**Figure 5:**
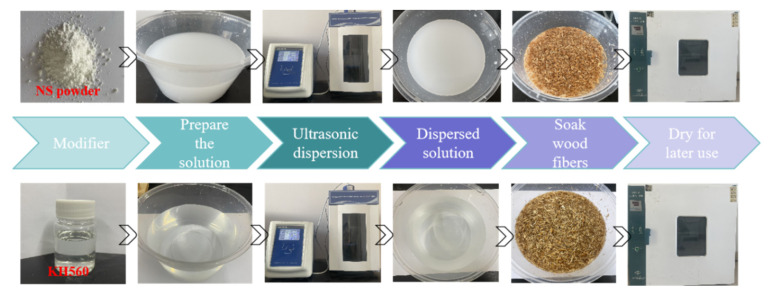
Modified process flow diagram of RWFs by NS and KH560.

**Figure 6:**
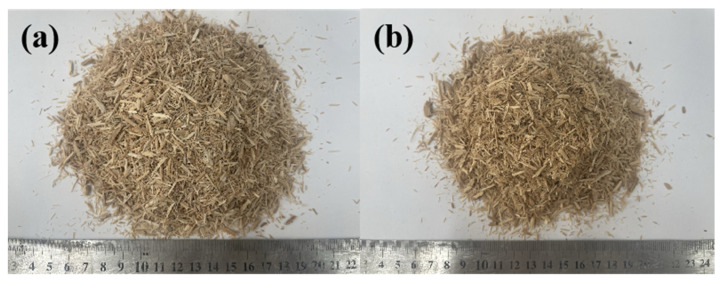
Appearance and morphology of different modified RWFs: (**a**) NS; and (**b**) KH560.

Regarding the ultrasonic dispersion conditions of the modification solution, the ultrasonic cell crusher used in this experiment was the model LC-JY16-IID produced by LICHEN Company, Shanghai, China. The power supply voltage of this instrument is 220 V, the maximum ultrasonic power is 1000 W, and the amplitude rod is Φ6 + Φ15. The modification method for the RWF is as follows: the prepared 1 wt% NS solution and KH560 solution are sufficiently stirred and then dispersed using ultrasonic dispersion; the solution-dispersing power and dispersing duration are set to 600 W and 30 min, ultrasonic on for 3.0 s and ultrasonic off for 2.0 s, to obtain the mass fraction of 1 wt% of the NS solution and KH560 solution. The RWFs were put into the dispersed solution and stirred and soaked. Then, the excess water was poured out and the RWFs were dried at 80 °C after three days of soaking. The specific modification process of the RWF is shown in Figure 5. The appearance and morphology of the different modified RWFs are shown in Figure 6.

**Figure 7:**
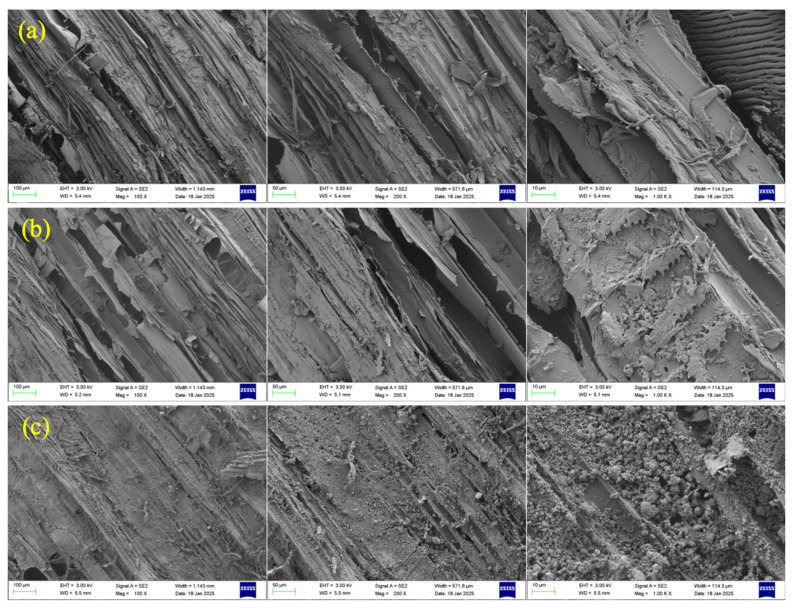
SEM images of modified RWFs: (**a**) unmodified RWF; (**b**) modified RWF with KH560; and (**c**) modified RWF with NS.

The SEM microscopic morphology of the modified RWFs is shown in Figure 7. As can be seen from the figure, the surface of the unmodified RWF is relatively smooth and regular, with a clear longitudinal texture, which is a typical feature of natural RWFs. The RWFs show a relatively tightly arranged structure. Compared with unmodified RWFs, the surface of KH560-modified RWFs becomes rough; this modification may increase the active sites on the fiber surface and change the chemical properties of the fiber surface, so that the interfacial compatibility with the substrate may be improved. NS modification presents a more pronounced silica nanoparticle attachment layer; the fiber surface is covered by a large number of silica nanoparticles covering the fiber surface, forming a rougher and more complex surface structure. The different modifications altered the roughness, chemical properties, and microstructure of the surface of the RWFs, which can optimize the performance of composites by regulating the fiber interface.

#### 2.1.5. Water-Reducing Agents

The water-reducing agent was provided by Shandong Construction Science and Technology Research Institute Co., Ltd., Jinan, China. The test mixing water was ordinary tap water. The water-reducing agent used in this test was a polycarboxylic acid water-reducing agent (PCE), the water-reducing rate was about 20% and the dosage of PCE is 2% of the amount of cementitious material.

### 2.2. Mix Proportion Design of RWF-SAC Composites

In this study, rapid-hardening sulfoaluminate cement was used as the cementitious material system, the cement–sand ratio was designed as 1:1, and the water–cement ratio was set at three gradients of 0.45, 0.50, and 0.55, respectively. With 0% RWF adding as the control group, different RWF contents of 10%, 20% and 30% were designed to systematically study the influence of different modification methods on SAC-RWF cementitious materials. The dosage of PCE is 2% of the SAC cement content, which remains constant under different water–cement ratio conditions. The ratios of RWF reinforced SAC cementitious materials under different modification methods are shown in Table 3, where “NWF” refers to nano-silica modified RWF, “KWF” refers to silane coupling agent modified RWF, and “**-**” indicates different modified RWF content (%) water–cement ratios. For example, NWF20-45 presents a RWF-SAC composites with 10% NS-modified RWF content at water–cement ratio of 45%.

### 2.3. Experimental Methods

The 28 d compressive and flexural strengths of RWF-SAC cement-based composites under different modifications were determined using a Type DYE-300S fully automatic cement flexural-compressive testing machine (Rongjida Instrument Technology Co., Ltd., Shanghai, China), with a span of 100 mm at loading rates of 50 N/s (flexural) and 0.6 kN/s (compressive), averaging six specimens per mix proportion. At 28 d of curing, three specimens per formulation were dried to constant mass at 40 °C in a 101 electric blast drying oven (Yongguangming Medical Instrument Co., Ltd., Beijing, China); dry density was calculated from mass-to-volume ratios. The dried test specimens in were placed water and the change in water absorption was measured over time to indicate the water absorption rate. The calculation method for the water absorption of fibers first involves subtracting the sample mass before water absorption from the mass after water absorption. Then, the result is divided by the mass before absorption. Finally, this is multiplied by 100% to obtain the final water absorption rate. Shrinkage values at 3, 14, 28, 45, and 91 days were measured using a Type LSP-1000S vertical mortar shrinkage–expansion instrument (Leicht Instrument Equipment Co., Ltd., Shenzhen, China). For thermal conductivity, 300 mm × 300 mm × 35 mm specimens were cured under (23 ± 2) °C and (50 ± 10)% RH for 28 days, dried to constant mass, and tested using an IMDRY3001-II dual-plate thermal conductivity meter (Chengsi Intelligent Technology Co., Ltd., Shanghai, China) with cold/hot plates set at 15 °C/35 °C. Hydration heat characteristics were monitored via a TAM Air eight-channel microcalorimeter, phase composition was analyzed by XRD (Bruker D8 ADVANCE, Bruker Corporation, Ettlingen, Germany, scanning angle 5–60°) and TG-DSC testing ( TGA/DSC 3+,METTLER TOLEDO, Zurich, Switzerland,), while microstructures and hydration products distribution were characterized using a SEM5000X field-emission SEM (CIQTEK, Hefei, China).

## 3. Results and Discussion

### 3.1. Mechanical Properties of Different Modified RWF-SAC Composites

#### 3.1.1. Flexural Strength of Modified RWF-SAC Composites

**Figure 8:**
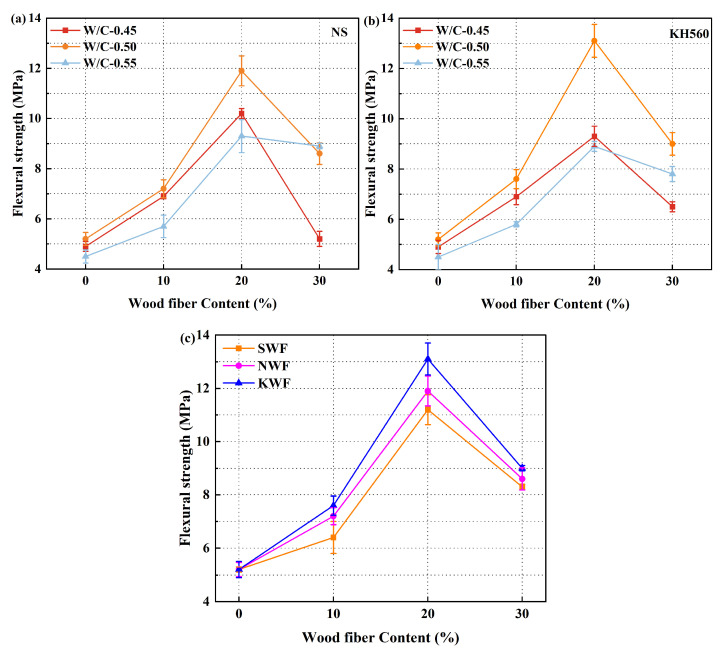
Flexural strength at 28 d of different modified RWF-SAC materials: (**a**) NS-modified series at different water–cement ratios; (**b**) KH560-modified series at different water–cement ratios; and (**c**) comparison of different modification methods (W/C-0.50).

Figure 8 presents the 28-day flexural strength evolution of modified RWF-SAC composites. As illustrated in Figure 8, the flexural strength demonstrates a characteristic pattern of initial enhancement followed by gradual reduction with increasing RWF content, indicating a non-linear relationship between fiber loading and mechanical performance. The optimal flexural strength is achieved at 20% RWF content, suggesting a synergistic interaction between fiber proportion and water–cement ratio. Notably, Figure 8a,b demonstrates superior flexural performance at a water–cement ratio of 0.50. The comparative analysis in Figure 8c further reveals that modified RWFs significantly enhance mechanical properties at this optimal ratio. Specifically, NS-modified and KH560-modified SAC composites exhibit respective strength improvements of 3.6% and 8.5% compared to the unmodified control group. Nano-silica particles mainly form physical coatings on RWF surfaces through van der Waals forces, hydrogen bonding, and electrostatic interactions. This surface roughening effect promotes mechanical interlocking with the cementitious matrix. In contrast, for the KH560-modified group, hydrolyzed silanol groups (-Si-OH) undergo condensation reactions with hydroxyl groups on RWF surfaces, establishing stable Si-O-C covalent bonds. Concurrently, the organofunctional termini of silane molecules develop chemical/physical interactions with the polymeric matrix, thereby optimizing interfacial adhesion. These findings confirm that both modification methods effectively enhance the mechanical performance of RWF-SAC composites, with KH560 demonstrating greater enhancement due to its covalent bonding mechanism.

#### 3.1.2. Compressive Strength of Modified RWF-SAC Composites

**Figure 9:**
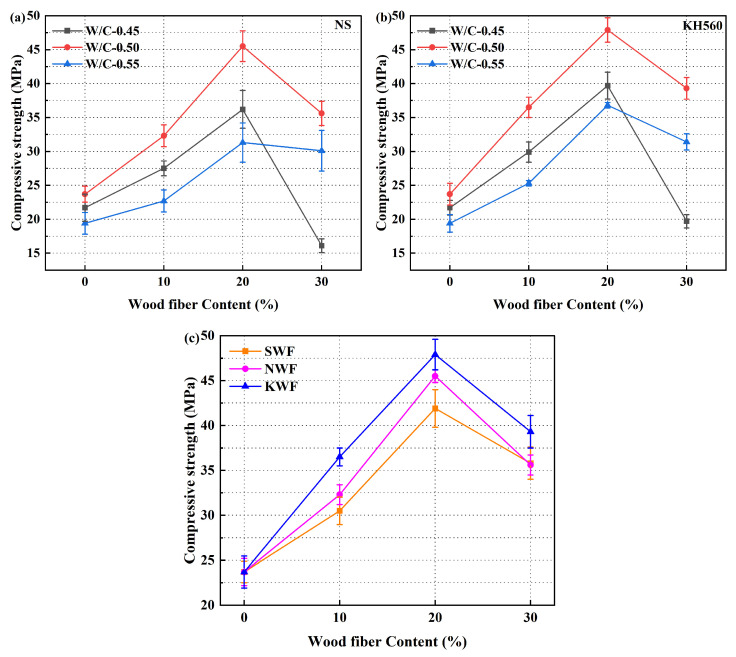
Compressive strength at 28 d of different modified RWF-SAC materials (**a**): NS-modified series at different water–cement ratios; (**b**): KH560-modified series at different water–cement ratio; and (**c**): comparison of different modification methods (W/C-0.50).

Figure 9 shows the 28-day compressive strength evolution of RWF-SAC composites under different modification methods, exhibiting a consistent trend with flexural strength behavior wherein compressive strength initially rises then declines with increasing RWF content, peaking at a water–cement ratio of 0.50. Compared to unmodified controls, NS and KH560-modified composites achieved enhanced compressive strengths of 45.5 MPa (8.6%) and 47.9 MPa (14.3%), respectively, attributable to dual mechanisms: NS modification elevates fiber surface roughness by particle adsorption due to van der Waals/hydrogen bonding while concurrently accelerating cement hydration to refine pore structure and reduce porosity [46], whereas KH560 modification enhances fiber dispersion in cement paste and establishes covalent Si-O-C bonds through silanol-hydroxyl condensation, synergistically strengthening interfacial adhesion and hydration efficiency. The inferior performance of NS-modified systems likely stems from increased fiber water absorption caused by NS surface deposition. These results conclusively demonstrate the superior enhancement of KH560 modification, yielding a 14.3% compressive strength improvement than that of the control groups, attributable to its chemical bonding mechanism and optimized fiber–matrix integration.

### 3.2. Dry Density of Modified RWF-SAC Composites

**Figure 10:**
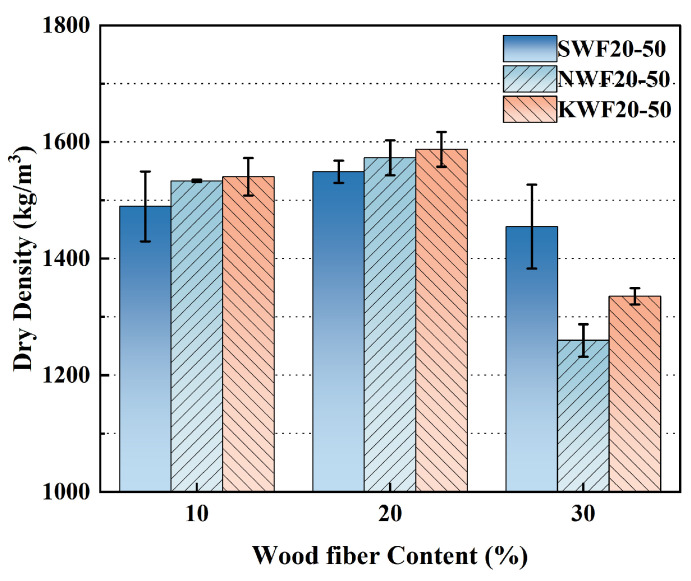
Dry density curve of different modified RWF-SAC materials at W/C ratio of 0.5.

Figure 10 shows the dry density evolution of RWF-SAC cementitious composites under different modification methods, revealing enhanced densification across both modified systems. For NS-modified specimens, the density elevation stems from nano-silica adsorption on fiber surfaces via van der Waals/hydrogen bonding interactions, which concurrently stimulates cement hydration to produce more hydration product formation while enabling nanoparticle infiltration into interfacial pores and intrinsic cementitious microvoids, thereby refining cement paste compactness. In contrast, KH560-modified systems achieve superior densification through silane hydrolysis-derived silanol groups that covalently bond with fiber hydroxyls (-OH) via Si-O-C linkages [47], effectively minimizing interfacial voids and fiber agglomeration while optimizing stress transfer across the interface. This dual-phase enhancement mechanism ensures tighter fiber encapsulation within the cement matrix, suppresses defect-induced porosity, and promotes complete hydration, collectively driving the increment of dry density. Notably, the KH560 modification method demonstrates greater density enhancement efficiency compared to NS-based modifications, aligning with the mechanical performance trends.

### 3.3. Thermal Conductivity of Modified RWF-SAC Composites

**Figure 11:**
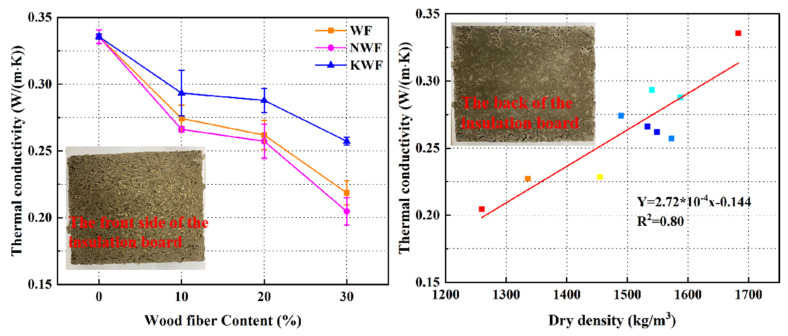
Thermal conductivity and linear regression curves of different modified reclaimed wood fiber reinforced cement-based materials.

Figure 11 shows the thermal conductivity curves of the RWF-SAC cementitious materials under different modification methods. It can be seen that the thermal conductivity of different modified RWF-SAC cementitious materials show a decreasing trend with the increase in RWF content. This indicates that the increase in the RWF content reduces the thermal conductivity of the RWF-SAC materials, which leads to the enhancement of their thermal insulation properties. For different modification methods, NS modification decreased the thermal conductivity density of cementitious materials by 6.8% compared to that of the control group, while the thermal conductivity increased for the KH560-modified series, which is consistent with the results of the mechanical properties and dry density; the modified RWFs improved the interfacial bonding properties with the cementitious matrix, resulting in the strength, dry density, and thermal conductivity of the cementitious materials. The dry density and thermal conductivity of the cementitious materials were all increased. The linear regression curve shows a good linear correlation between dry density and thermal conductivity, R^2^ = 0.80, indicating that the linear regression model fits the data well with moderate linear correlation. Overall, due to the increase in dry density and mechanical properties of KH560-modified RWF-SAC materials, the thermal conductivity also increased; however, the thermal conductivity remained at a low level.

### 3.4. Water Absorption of Modified RWF-SAC Composites

**Figure 12:**
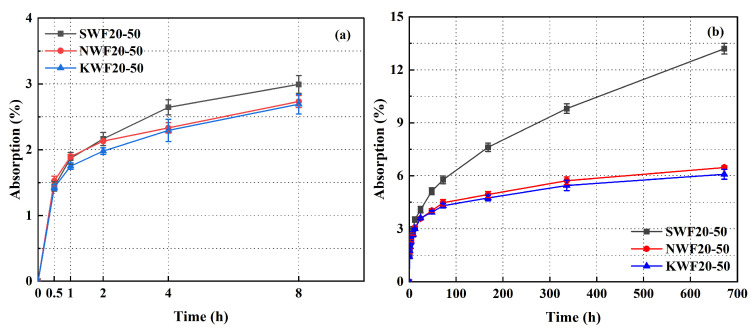
Water absorption curves of different modified RWF-SAC materials: (**a**) early age; and (**b**) long-term age.

Figure 12 shows the water absorption curves of RWF-SAC materials under different modification methods. The water absorption rate of 672 h (28 d) was determined in the test; different modifications had basically the same water absorption rate for 0.5 h. The water absorption rate of the KH560-modified series began to slow down from 0.5 h onwards. The water absorption rate of the modified regenerated wood fiber and cementitious materials was greatly reduced compared to that of the control group; the KH560 series had the lowest water absorption rate, which had a remarkable effect. The water absorption of NS series and KH560 series cementitious materials were 6.47% and 6.09%, respectively, which were 6.72% and 7.1% lower than those of the control group. For the NS series, NS can promote the hydration reaction of cement and fill the pores, which reduces the pore size and decreases the porosity in cementitious materials, thus reducing the water absorption rate. As for the KH560 series, the silane coupling agent improves the hydrophobicity of the recycled wood fibers by chemically bonding with the surface of the recycled wood fibers [48], which makes the water absorption of the recycled wood fibers decrease; most of the increase in water absorption in the later stage is due to the high water absorption characteristics of the recycled wood fibers. The recycled wood fibers can be saturated with water earlier after the modification of the silane coupling agent, so that the growth of the water absorption rate in the 72 h will be slow. The effect of the silane coupling agent on the water absorption rate of RWF-SAC material is more obvious and the reduction in the water absorption rate has a positive effect on the durability of the RWF-SAC cementitious materials.

### 3.5. Shrinkage Properties of Modified RWF-SAC Composites

**Figure 13:**
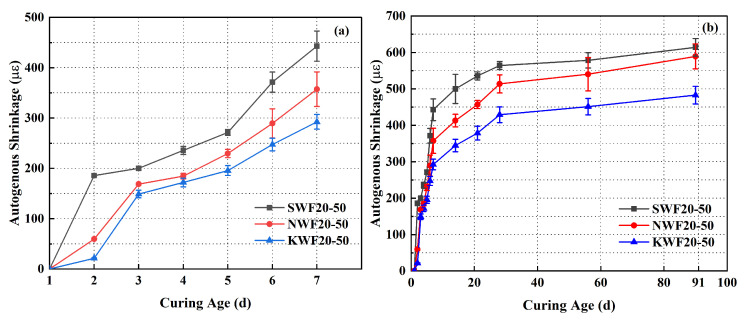
Shrinkage properties of different modified RWF-SAC materials: (**a**) 7-days; (**b**) 91-days.

Figure 13 shows the shrinkage performance curves of the RWF-SAC materials under different modification methods. From Figure 13a,b, it can be seen that the modified series had a reduced self-shrinkage value than that of the unmodified control group. This indicates that the modified wood fibers can further resist the drying shrinkage of cement paste. The self-shrinkage process of the material mainly occurs in the first 7 d; the growth slows down after 7 d, which is related to the hydration reaction of the cement. The modified series of KH560 showed lower shrinkage values at this stage, which indicates that the modifier acts at an early stage and significantly reduces the shrinkage of the material. As the time extended to 91 days, the shrinkage values of unmodified and modified series cement-based composites tended to flatten out. The silane coupling agent enhanced the interfacial bonding between the fibers and the cement matrix through chemical bonding, reducing water loss and internal stress concentrations. The interfacial reinforcement may reduce the risk of micro-crack extension and thus inhibit the self-shrinkage. NS particles fill the micropores of the cement matrix to improve the densification and reduce the shrinkage caused by water loss from the capillary pores. However, the effect was weaker than that of KH560-modified series, probably because it did not directly improve the fiber–matrix interface. However, it filled in the pores of the cement paste, making its improvement effect weaker than that of KH560. In summary, the KH560 modification was the most effective, reducing the self-shrinkage by about 27.2% compared with the control group. The NS modification was the second most effective, reducing the shrinkage by around 4.3%.

### 3.6. Microscopic Properties of Modified RWF-SAC Composites

#### 3.6.1. Hydration Characteristics of Modified RWF-SAC Composites

**Figure 14:**
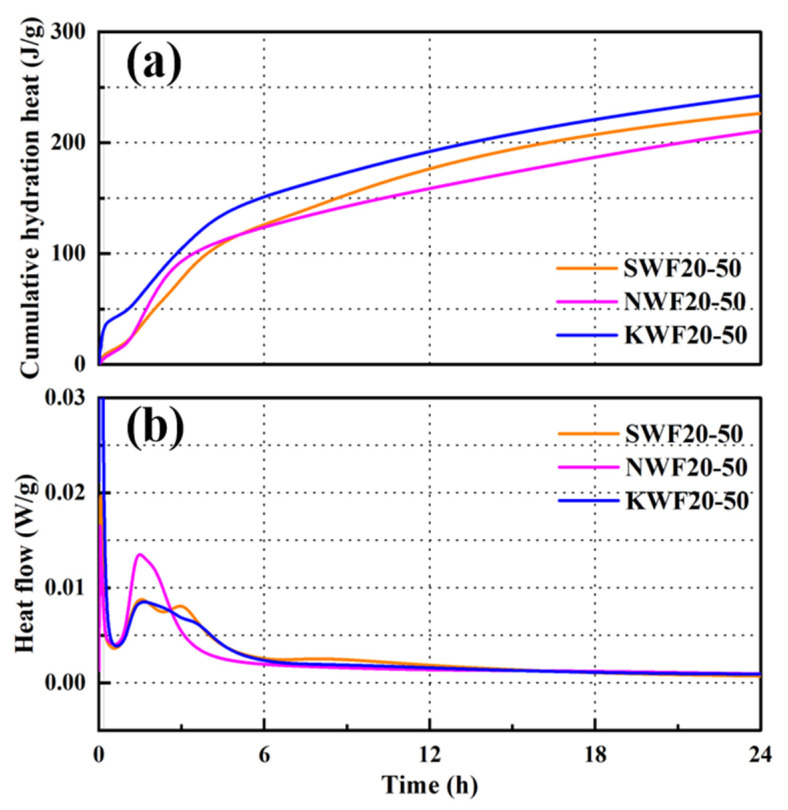
Hydration heat curves of different modified RWF-SAC materials: (**a**) Cumulative hydration; (**b**) Heat flow.

Figure 14 presents the hydration exothermic curves of RWF-SAC materials incorporating different modified RWFs. The cumulative heat release of paste reflects the degree of paste hydration, while the heat flow curve clearly illustrates each stage of the hydration process. As shown in Figure 14a, the KH560-modified series exhibits rapid early-stage hydration reactions of SAC materials, with significantly higher final cumulative hydration heat than that of the control group. The NS-modified series demonstrates accelerated heat release within 6 h, surpassing the control group in cumulative hydration heat, although its curve growth gradually slows in later stages, indicating a decelerating hydration rate. This suggests that different modification methods of RWFs variably influence the cement hydration of SAC composites, with KH560 modification potentially being more conducive to sustained hydration reactions, thereby releasing greater cumulative heat of hydration.

From Figure 14b, all three series display distinct heat flow peaks representing the maximum cement hydration rates. Compared to the control group, the NS-modified series achieves the highest peak heat flow, indicating its momentarily faster early-stage reaction rate. This may be attributed to the uniform dispersion of NS particles in the cement matrix, promoting rapid growth of hydration products, like aluminum gels, and releasing additional heat. In contrast, the KH560-modified series exhibits a lower early-stage peak heat flow due to the organic film formed on the fiber surface, which temporarily hinders water–cement contact and slightly reduces initial reaction rates. As hydration progresses, KH560 enhances the interfacial bonding between fibers and the cement matrix, facilitating the growth and accumulation of hydration products at the interface. This promotes more thorough hydration, improving later-stage reaction degrees and ultimately yielding higher cumulative heat flow than the NS-modified series. Thus, the KH560 modification exerts a more substantial influence on cement hydration, enabling more complete reactions that release more heat.

#### 3.6.2. Thermogravimetric Analysis of Modified RWF-SAC Composites

**Figure 15:**
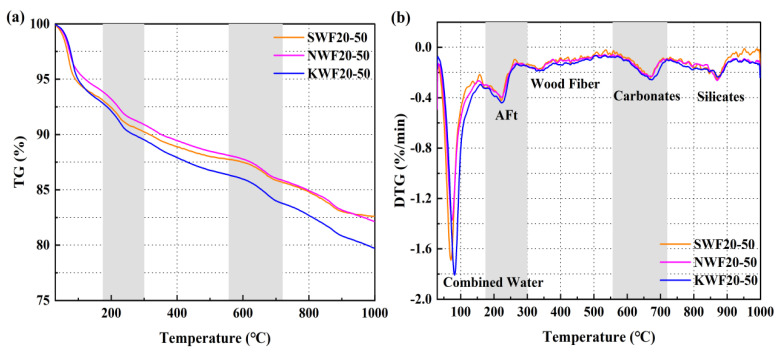
TG-DSC curves of different modified RWF-SAC materials: (**a**) TG; (**b**) DSC.

Figure 15 shows the TG-DSC curves of different modified RWF-SAC materials. It can be seen that the rate and extent of mass loss at the initial stage (<180 °C) varied slightly among the three different modified series. With the 180–300 °C range for calcite decomposition, the peaks of calcite decomposition from the highest to the lowest are the KH560-modified series, the NS-modified series, and the control group, respectively. Since the amount of calcite and gel can affect the strength and durability of cementitious materials, the hydration characteristics and macro performance are closely related. The decomposition peak of wood fiber at about 300 °C is basically the same due to the same content. The decomposition peak of CaCO_3_ at 560–720 °C is consistent with the decomposition peak of bound water and ettringite (AFt), which are representative of the strength of materials. The decomposition peak of silicate, at about 900 °C, is the same. The size of the peaks at this time is in the following order: NS series, control, and KH560 series. The height of the calcium silicate decomposition peak indicates how much unhydrated silicate is in the cement; therefore, the KH560 series is more completely hydrated than the NS series. The analysis of the TG-DSC curves of different modified recycled wood fiber cementitious composites showed that NS modification and KH560 modification had some effects on the removal of bound water, decomposition of ettringite and silicate in the composites. The highest weight loss in the cement paste after KH560 modification represented the most obvious effect of promoting the hydration reaction but it had less of an effect on the pyrolysis process of the recycled wood fibers and the decomposition of the carbonates.

#### 3.6.3. XRD Analysis of Modified RWF-SAC Composites

**Figure 16:**
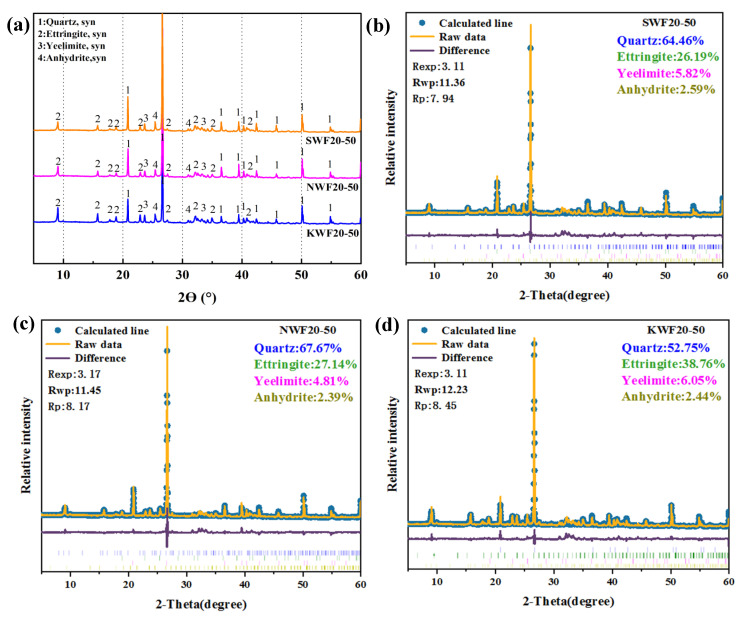
XRD patterns and quantitative analysis of different modified RWF-SAC materials: (**a**) XRD patterns of different modified materials; (**b**) SWF20-50; (**c**) NWF20-50; and (**d**) KWF20-50.

Figure 16 shows the XRD curves of the hydration products of different RWF-SAC materials. From Figure 16a, it can be seen that the modification treatment of the RWF did not cause the SAC cementitious materials to generate new hydration products; however, it had a certain effect on the content of the hydration products. The physical phases shown by the XRD patterns are mainly quartz, ettringite, calcium silica-aluminate, and gypsum. From the figure, it can be seen that, compared to the control group, the peaks of the NS-modified and KH560-modified series of calcium alumina are relatively high, which is consistent with the TG-DSC thermogravimetric results. In addition, the graph shows the amount of gypsum content in the order of the control, NS-modified, and KH560-modified series. The analysis of the XRD curves shows that NS modification and KH560 modification have less influence on the type and crystal structure of the main mineral phases of the recycled wood fiber cementitious materials but the degree of hydration of the cement can be reflected by the change in the relative content. Figure 16b–d shows the XRD quantitative analysis of different hydration products. Due to the direct introduction of nano-SiO_2_ in NS modification, the quartz peak intensity is significantly increased, to 67.67%. The unmodified SWF group ranks second, despite KH560 containing Si-forms amorphous Si-O-C bonds (invisible to the XRD pattern), resulting in a relatively lower quartz phase content of only 52.75%. Simultaneously, KH560 enhances the wood fiber–cement interfacial bonding, reducing competitive water absorption and promoting stable ettringite formation, yielding the highest ettringite content of 38.76%. NS particles primarily fill pores, leading to the similar ettringite peak values between NS-modified and SWF groups, with only minor improvements. The amount of gypsum content represents the magnitude of cement hydration; the lower the gypsum content, the more thoroughly the hydration of the cementitious material is carried out. As gypsum serves as the precursor for AFt formation, accelerated hydration in modified groups consumes more gypsum. Both the KH560 and NS groups exhibit substantial gypsum consumption, whereas the unmodified group shows the highest gypsum residue due to delayed hydration. Compared with NS modification, the interfacial bond between wood fiber and cement matrix was enhanced after KH560 silane coupling agent modification, which provided more favorable conditions for the formation and growth of hydration products. At the interface, the cement hydration products can grow and accumulate in a more orderly manner, forming a denser interfacial transition zone. This means that the modification effect may be more in improving the interfacial bonding properties between the fiber and the paste, reinforcing the mechanical properties of the material, rather than playing a role by changing the mineral phase composition of the SAC cementitious material.

#### 3.6.4. SEM Microscopic Morphology of Modified RWF-SAC Composites

**Figure 17:**
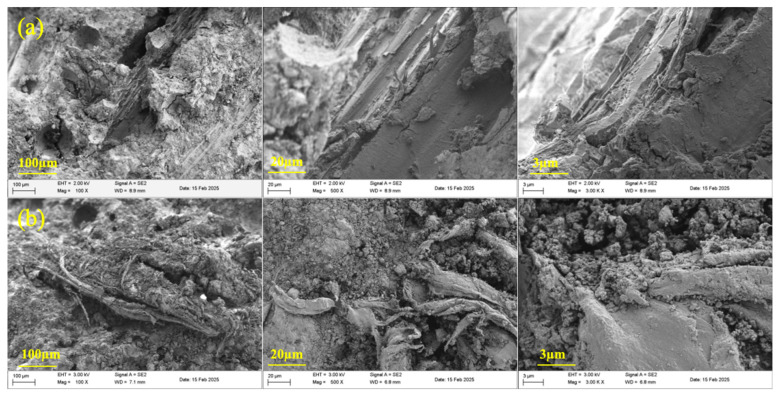
SEM micro-morphologies of different modified RWF-SAC materials: (**a**) KWF20-50; and (**b**) NWF20-50.

Figure 17 shows the SEM micrographs of RWF-SAC cementitious materials under different modification methods, comparing the microstructures of the two modified series at magnifications of ×100, ×500, and ×3000. It can be seen that in the KH560-modified series, RWF is primarily embedded within the SAC cement matrix, whereas in the NS-modified series, it adheres to the surface of RWF. This indicates superior adhesion between KH560-modified RWF and the SAC cement paste. As magnification increases, the interfacial transition zone (ITZ) between RWF and the cement matrix becomes more distinct. Most KH560-modified RWF are observed embedded within hydration products, while NS-modified RWF remains largely exposed with many microcracks, suggesting stronger interfacial bonding in the KH560-modified composite. At higher magnification (×3000), the microstructure of SAC hydration products and their interaction with RWF become clearer. Notably, the surface of NS-modified RWF is covered with numerous fine particles, likely a mixture of silica nanoparticles and cement hydration products. The presence of nano silica particles increases surface roughness and active sites, enhancing mechanical interlocking and chemical bonding between the fiber and cement matrix.

**Figure 18:**
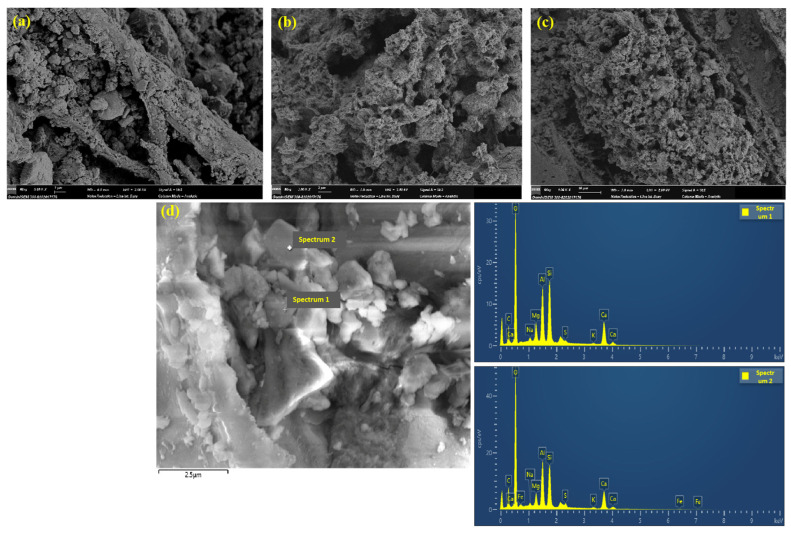
SEM images of NS-modified RWF-SAC (NWF20-45): (**a**) ×5000; (**b**) ×3000; (**c**)×1000; and (**d**) SEM-EDS analysis of (**c**).

**Figure 19:**
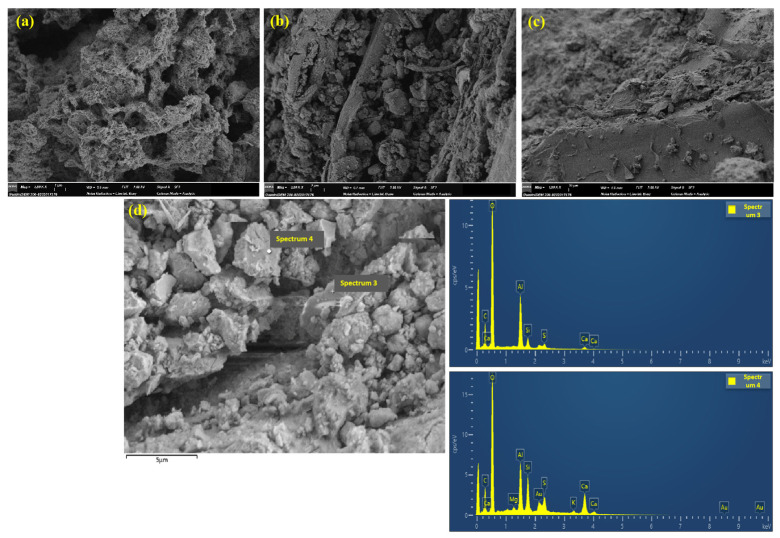
SEM images of KH560-modified RWF-SAC (KWF20-45): (**a**) ×5000; (**b**) ×3000; (**c**) ×1000; and (**d**) SEM-EDS analysis of (**i**).

Figure 18a–c shows the SEM microstructures of NS-modified RWF-SAC (NWF20-45). Numerous NS particles are attached to the surface of RWF, with discernible pores existing between the RWF and cement paste. Some NS particles exhibit flocculation and agglomeration. Figure 18d presents the corresponding SEM-EDS analysis of Figure 18c. Strong Si element signals are detected at the interface and pores, with Si elements showing clustered enrichment around pores and fibers. As the Si element is the primary component of nano-SiO_2_ particles, this confirms that NS particles fill the fiber-cement interface or matrix pores, indicating that they enhance the density of the composite cementitious material through physical filling and nano adsorption mechanisms. Figure 19a–cshows the SEM microstructures of KH560-modified RWF-SAC (KWF20-45). In contrast, the fracture surface of the KH560-modified composite reveals abundant fibrillated structures. This phenomenon likely results from the high interfacial bond strength between the cement matrix and modified fibers. During specimen failure, fibers absorb significant energy and undergo internal fracture, producing these fibrillated features. Figure 19d presents the SEM-EDS analysis of Figure 19c. Point scanning at the fiber–cement contact zone shows the coexistence of C (from RWF) and Si (from KH560) at the interface, with enhanced O element peaks bridging the spectra. This confirms the formation of Si-O-C chemical bonds on the RWF surface due to the presence of KH560. The synergistic enrichment of Si, C, and O at the interface, along with their gradient distribution, indicates chemical bonding. Concurrently, the diffusion of Ca and Al elements appears weakened near the fiber interface, likely due to steric hindrance from the chemical bonds.

In summary, the SEM microscopic analysis under different magnifications reveals that both the silane coupling agent (KH560) and nanosilica (NS) modifications improve the compatibility and interfacial properties of different RWF-SAC materials. However, the KH560 modification method proves more effective in strengthening interfacial adhesion and optimizing the microstructure of RWF-SAC composites. These micro-structural variations ultimately influence the mechanical properties and macroscopic performance of the RWF-SAC cement-based composites.

## 4. Conclusions

In this study, the effects of both nano-silica (NS) modification and silane coupling agent (KH560) modification on the hydration and performance of recycled wood fiber (RWF) reinforced SAC cementitious composites were investigated at different water–cement ratios and RWF content, to enhance the interfacial bonding performance and overall macroscopic performance. The main conclusions obtained are as follows:

(1) The modified RWF-SAC cementitious composites demonstrate optimal mechanical performance at a water–cement ratio of 0.50 with 20% RWF content. Comparative analysis of differently modified RWF-SAC cementitious materials reveals that both NS and KH560 modifications enhance compressive strength, with KH560 exhibiting more pronounced effects, achieving 8.5% and 14.3% improvements in flexural and compressive strength, respectively, compared to the unmodified control group;
(2) The modified RWF-SAC cementitious composites exhibited a strong linear correlation (R^2^ = 0.80) between dry density and thermal conductivity. The experimental results demonstrated that the thermal conductivity of different modified RWF-SAC cementitious materials showed a decreasing trend with the increase in RWF content. While NS-modified samples showed a 6.8% reduction in thermal conductivity compared to the control group, the KH560-modified series displayed increased thermal conductivity while maintaining relatively low overall values;
(3) Regarding the shrinkage performance, both modification methods significantly improved water absorption. The water absorption of NS and KH560 series cementitious materials were 6.47% and 6.09%, respectively, which were 6.72% and 7.1% lower than those of the control group. Shrinkage resistance was also markedly enhanced, as the KH560 modification reduced autogenous shrinkage by approximately 27.2%, representing the most effective improvement among all samples;
(4) In terms of hydration characteristics, through the comparison of hydration characteristics of cementitious materials with different modified recycled wood fibers, it was found that the KH560-modified series had a relatively larger effect on cement hydration heat and could make cement hydration more adequate. The NS-modified series mainly improved the reflection rate during cement hydration, which might be related to the ability of NS particles to promote cement hydration;
(5) XRD patterns and SEM analysis show that the modified treatment of RWF does not make the cementitious materials generate new hydration products but has a certain effect on the hydration product content. The KH560-modified RWF-SAC materials have better interfacial bonding properties, generating more hydration products, the porosity is significantly reduced, the internal structure is more dense, and the overall modified effect of the KH560 series is better than the NS modification series.

## Figures and Tables

**Table 1:** The mineral composition of rapid-hardening sulphoaluminate cement (%).

Chemical Composition	CaO	Al_2_O_3_	SO_3_	SiO_2_	MgO	Fe_2_O_3_	TiO_2_	L.o.I.
Proportion	51.5	22.47	8.93	8.77	3.88	3.69	0.34	0.42

**Table 2:** Physical performance indicators of natural river sand.

Fine Aggregate	Voidage/%	Micro-Powder Content/%	Mud Content/%	Crushing Index/%
Natural river sand	37	0.9	0.8	11.2

**Table 3:** Mix proportions of modified RWF-SAC cement-based materials.

Group Number	Water–Cement Ratio	The Amount of RWFs /%	Modification Method	Raw Material/g				
				Sand	RWF	Cement	Water	PCE
NWF0-45	0.45	0	NS	800	0	800	360	8
KWF0-45			KH560					
NWF10-45		10	NS	700	70	700	315	7
KWF10-45			KH560					
NWF20-45		20	NS	600	120	600	270	6
KWF20-45			KH560					
NWF30-45		30	NS	500	150	500	225	5
KWF30-45			KH560					
NWF0-50	0.50	0	NS	800	0	800	400	8
KWF0-50			KH560					
NWF10-50		10	NS	700	70	700	350	7
KWF10-50			KH560					
NWF20-50		20	NS	600	120	600	300	6
KWF20-50			KH560					
NWF30-50		30	NS	500	150	500	250	5
KWF30-50			KH560					
NWF0-55	0.55	0	NS	800	0	800	440	8
KWF0-55			KH560					
NWF10-55		10	NS	700	70	700	385	7
KWF10-55			KH560					
NWF20-55		20	NS	600	120	600	330	6
KWF20-55			KH560					
NWF30-55		30	NS	500	150	500	275	5
KWF30-55			KH560					

## Data Availability

Data are contained within the article.
